# Electrolytic Recovery of Metal Cobalt from Waste Catalyst Pickling Solution

**DOI:** 10.3390/ma15196629

**Published:** 2022-09-24

**Authors:** Yi-Sin Chou, Chin-Hsiang Kan, Nitika Devi, Yong-Song Chen

**Affiliations:** 1 Chemical Engineering Division, Institute of Nuclear Energy Research, Longtan, Taoyuan 325207, Taiwan; 2 School of Physics and Material Sciences, Shoolini University, Solan 173229, India; 3 Department of Mechanical Engineering and Advanced Institute of Manufacturing with High-tech Innovations, National Chung Cheng University, Minhsiung Township, Chiayi 621301, Taiwan

**Keywords:** terephthalic acid, electrolyzer, electrochemical reduction, cobalt recovery

## Abstract

Terephthalic acid production plant uses liquid cobalt–manganese bromide as a catalyst. The waste catalyst is burned with exhaust gas and accumulated in fly ash, which is further pickled and impregnated with a sulfuric acid solution. The resultant solution is rich in cobalt and manganese metal ions with few metal impurities from other petroleum raw materials. An electrochemical reduction method is used to recover cobalt metal from the waste catalyst fly ash pickling solution of terephthalic acid. Various steps have been taken to remove impurities and extract and separate the required pure cobalt metal solution. Afterward, the process of electrolytic reduction smelting is conducted. Variables investigated include current density, electrolyte pH, electrode materials, and electrolytic cell diaphragms, among several others. Results show that the product purity can reach up to 99.84% for the electrolyte feed composition of 21.4 g L^−1^ Co, 38.2 g L^−1^ Na, and 2.02 g L^−1^ Mg.

## 1. Introduction

Transition metals, among all periodic elements, have special importance due to their global average recycled content [1] and wide applications. Among them, cobalt (Co) and nickel (Ni) are widely used in metallurgy, chemical industry, high-temperature magnetic alloys, battery, and mining industries [2]. Cobalt metal is crucial in the industrial development and economic life of humans. It provides various structural materials and is an indispensable element for the petrochemical industry since it functions as a conversion catalyst. The United States established the Madison Project to be self-sufficient using superalloy-grade cobalt materials after the strategic material crisis in 1978. Canada has the largest material reserves in North America. The production of cobalt and nickel in 2019 was approximately 180,000 tons and 3000 tons, respectively [3]. This mainly involved the mined cobalt pentlandite {(Fe, Ni, Co)_9_S_8_} and linnaeite (Co_3_S_4_). In addition, cobalt and nickel elements [4] are rich in sulfide ores such as pentlandite {(Ni, Fe)_9_S_8_}, millerite (NiS), and violarite (Ni_2_FeS_4_) [5]. From 2015 to 2025, it has been established that lithium-ion batteries (LIBs) have higher voltage and energy density capabilities than other batteries. These batteries are more widely used in portable electronic devices, such as laptops, smartphones, and electric vehicles [6,7,8,9]. Cobalt is an important element that is used as a cathode material [9]. It is estimated that by 2030, Canada, Mexico, and the United States will produce about 676,000 electronic-drive vehicles that will incorporate LIBs. Demand for LIBs has surged by more than 13 times since 2015 [10]. In addition, cobalt is an essential element for various vehicle engines and is considered a strategic metal [7]. It is estimated that by 2050, the demand for cobalt and nickel will increase significantly [11], and the supply risk of cobalt will reach a critical level [12]. The global demand for nickel will also double or triple by 2050. Experts believe that the two elements will be present in only 30% of their demand. The demand can be fulfilled by recycling wastes containing metal elements [13,14].

The market demand for cobalt continues to grow, and research on its extraction is increasing. Cobalt and other metals exist simultaneously in various primary and secondary substrates such as nickel laterite and waste batteries [2]. Cobalt is leached from nickel laterite using chelating resin and solvent extraction techniques [15,16,17]. To recover cobalt from solid waste, the solids are pickled and impregnated first and must sometimes be roasted in advance for the metal to become an oxide that can be easily pickled and dissolved. Solvent extraction and recovery of cobalt in waste lithium-ion batteries [12], as well as research on the use of hydrometallurgy to extract cobalt, have been reported [18,19]. Japan JX Nippon Mining & Metals Corporation has used dialkylphosphinic and carboxylic acids to extract cobalt from LIB leachate [12,20]. Nickelhütte Aue GmbH, a German recycling company, has multiple product settings for nickel–metal hydride batteries containing cobalt depending on the method [20].

Because cobalt metal is important and expensive, developing and researching methods for recovering resources from waste is beneficial. Das and Subbaiah investigated the electrolytic reduction of pure cobalt solution, and their conclusion suggested that the pH value should be controlled between 2 and 4 [21] to obtain a bright cobalt deposition coating. Dubrovsky and Evans used fluidized-bed electrodes to electrolytically reduce pure cobalt solution (cobalt concentration is 4.8–100 g L^−1^) [22]. Research results show that the applicable range of acid–base values can be extended to pH = 1–6. The terephthalic acid production plant uses liquid cobalt–manganese bromide as a catalyst and the waste catalyst is burned with exhaust gas and accumulated in fly ash. Afterward, the fly ash is pickled and impregnated with a sulfuric acid solution. This solution is rich in metal ions such as cobalt and manganese with few metal impurities from other petroleum raw materials. The components in the acid impregnation solution are complex. Steps for removing impurities and extracting the pure cobalt metal should be conducted separately. Further, the process of electrolytic reduction smelting was conducted. The obtained cobalt metal can be refined again to improve the purity of the product. This study obtains practical operating conditions and evaluates the feasibility of building a factory in the future. Therefore, the variables that must be tested include current density, electrolyte pH, electrode materials, and electrolytic cell diaphragms, among several others.

## 2. Experimental Section

### 2.1. Electrolyte Preparation and Analysis

The electrolyte consisted of fly ash and sulfuric acid and was provided by a terephthalic acid production plant. The pH value of the electrolyte was controlled by sulfuric acid (ACS reagent, 95.0–98.0%, Sigma-Aldrich^®^, St. Louis, MO, USA), sodium carbonate (ACS reagent, anhydrous, ≥99.5%, Sigma-Aldrich^®^), and ammonium hydroxide (ACS reagent, 28.0–30.0% NH_3_ basis, Sigma-Aldrich^®^) at selected levels. The composition, impurity analysis of the electrolyte, and change in cobalt concentration during the electrolysis process were analyzed using an inductively coupled plasma emission spectrometer (PS1000, Leeman Labs Inc., Hudson, NH, USA) or atomic absorption spectrometer (PinAAcleTM 900F, PerkinElmer^®^, Waltham, MA, USA). In addition, an energy dispersive X-ray spectrometer was used to analyze the impurity concentration and surface morphology after sampling and purifying the cobalt. Scanning electron microscopy (S4800, Hitachi^®^, Tokyo, Japan) was used to analyze the surface morphology of deposited cobalt.

### 2.2. Electrolyzer Design for the Extraction Reaction

**Figure 1:**
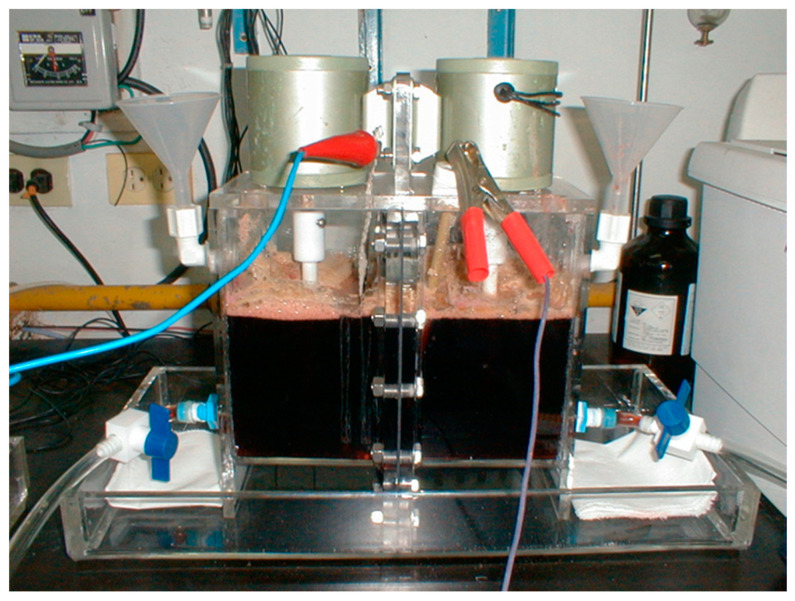
Electrolyzer used for extraction reaction.

The electrolytic cell used for the experiment was composed of acrylic, which is further divided into two parts: the anode and cathode chambers. These two parts were combined with screws to form a complete electrolytic cell. A piece of an organic diaphragm (NM-60, Ionac^®^) was used to separate the anode and cathode chambers to prevent electrolytes from mixing. The current transmission between the anode and cathode will depend on the energy and ions passing through the organic membrane. Figure 1 shows the electrolyzer used for the extraction reaction. The volume of the anode and cathode compartments was 2 L each. The electrolyte stirring was performed using a stirring rod installed above the electrolytic tank and a frequency converter controlled the stirring rate. The stirring rod is made of Teflon plastic material, which is resistant to acid and alkali.

The organic membrane in the middle of the electrolytic cell is such that only cations can penetrate the membrane. Because hydrogen ions transfer current the fastest, organic membranes are processed to allow for the transfer of hydrogen ions from the anode chamber to the cathode chamber. Cobalt metal electrolyte is an acidic electrolyte. Cobalt-containing metal electrolyte is placed in the cathode chamber and is affected by the negative electrode. Thus, potential cobalt ions will migrate to the negative electrode and deposit on the electrode plate. A 0.25 M amount of dilute sulfuric acid solution was added to provide protons to increase electrolyte conductivity. The cathode chamber is composed of a stainless-steel plate or titanium mesh, and the surrounding is sealed with tape. This is to prevent the current from concentrating on the sharp edges, and the center has an area of about 16 cm^2^. A platinum-plated titanium mesh anode with a size of about 10 × 12 cm^2^ (effective area of electrolysis) was used. Power was supplied via a DC power supply, which can adjust both voltage and current. The electrolyzer can also be operated without the organic resin diaphragm in the middle. The electrolyzer can now contain 4 L of cobalt-containing metal pickling solution. Other experimental conditions are the same as those chosen for the installed organic diaphragm electrolyzer. The ohmic resistance of the system is estimated to be about 0.9 Ω.

### 2.3. Current Efficiency

The cobalt metal obtained in the experiment was measured using the gravimetric method, and the theoretical weight of the cobalt metal was calculated using Faraday’s law of electrolysis. The actual weight of cobalt metal is divided by the theoretically strained weight of cobalt metal. The formula used for calculations is as follows:

(1) $$\eta =\frac{MnF}{M_{w}ti}\times 100\%\;$$

η: current efficiency (%);
M: mass of cobalt deposited on the cathode (g);
n: number of electrons transferred;
$M_{w}$: molecular weight (58.9 g mol^−1^ for cobalt);
*t*: electrolysis time (s);
*i*: current (A).

## 3. Results and Discussion

This study intends to recover waste liquid cobalt–manganese bromide catalyst from the terephthalic acid production plant, which is burned with exhaust gas and accumulated in fly ash. The electrochemical reduction method has been used for conducting this experiment. Table 1 shows the components and contents of the pickling solution. Due to the pickling test conditions, the concentration of the components varies slightly between batches. Mixed individual batch solutions have been used for subsequent experimental tests.

This study uses electrolytic reduction to recover cobalt metal. Because of the presence of trace metal impurities, the potential (*E*^0^) of ions contained in the electrolyte relative to the hydrogen reaction and possible reactions of the anode and cathode are presented as follows:

On the cathode side:

(2) $${\mathrm{Co}}^{2+}+2e^{-}\to \mathrm{Co}\;E^{0}=-0.28\;V$$

(3) $${\mathrm{Fe}}^{2+}+2e^{-}\to \mathrm{Fe}\;E^{0}\;=-0.44\;V$$

(4) $${\mathrm{Zn}}^{2+}+2e^{-}\to \mathrm{Zn}\;{E^{0}}_{\;}=-0.76\;V$$

(5) $${\mathrm{Ni}}^{2+}+2e^{-}\to \mathrm{Ni}\;E^{0}=-0.25\;V$$

(6) $${\mathrm{Pb}}^{2+}+2e^{-}\to \mathrm{Pb}\;E^{0}\;=-0.13\;V$$

(7) $$2H^{+}+2e^{-}\to H_{2}\;\;E^{0}=0.00\;V$$

(8) $${\mathrm{Cu}}^{2+}+2e^{-}\to \mathrm{Cu}\;\;\;E^{0}=0.34\;V$$

On the anode side:

(9) $$2H_{2}O\to O_{2}+4H^{+}+4e^{-}\;\;\;\;\;\;E^{0}\;=1.23\;V\;$$

(10) $${\mathrm{Mn}}^{2+}\to {\mathrm{Mn}}^{3+}+e^{-}\;\;\;\;\;\;\;\;{E^{0}}_{\;}=1.51\;V$$

(11) $${\mathrm{Co}}^{2+}\to {\mathrm{Co}}^{3+}+e^{-}\;\;\;\;\;\;\;\;\;\;{E^{0}}_{\;}=1.82\;V$$

(12) $${\mathrm{Mn}}^{2+}+2H_{2}O\to {\mathrm{MnO}}_{2}+4H^{+}+2e^{-}\;\;{E^{0}}_{\;}=1.22\;V$$

Because of the large concentration of Co^2+^ ions near the cathode, the aforementioned potential reactions occur mostly as a reduction process. However, the reduction potential is close to that of hydrogen ions, as predicted from Equation (7), thus it is the cathode’s main side reaction. It is necessary to suppress the reaction of Equation (7) and increase the possibility of the reaction of Equation (2) in the controlled electrolyte pH environment. Other impurities such as Ni^2+^, Cu^2+^, and Pb^2+^ are easier to reduce than Co^2+^, and the number of these metals in the solution affects the purity of metallic cobalt. In addition, Fe^2+^, Zn^2+^, and others are more difficult to reduce than Co^2+^ due to the limited potential difference. Particularly, Zn^2+^ ions have abnormal potential in acidic aqueous solutions, and their reducibility is higher than Ni^2+^ and Fe^2+^. In addition, they are easier to deposit on the cathode. Alternatively, the cation reaction is mostly Equation (9), which involves the production of sulfuric acid and oxygen. Although Equation (12) is similar to Equation (9) in terms of potential, electrolytic manganese dioxide has high activation energy. Therefore, this is possible only under higher temperatures. Equations (10) and (11) are less likely to occur in this potential than Equation (9), but Co^3+^ and Mn^3+^ are strong oxidants. If this side reaction occurs, it can accelerate the electrolytic oxidation of water, producing more oxygen and sulfuric acid.

### 3.1. Influence of Electrolyte pH on Current Efficiency

**Figure 2:**
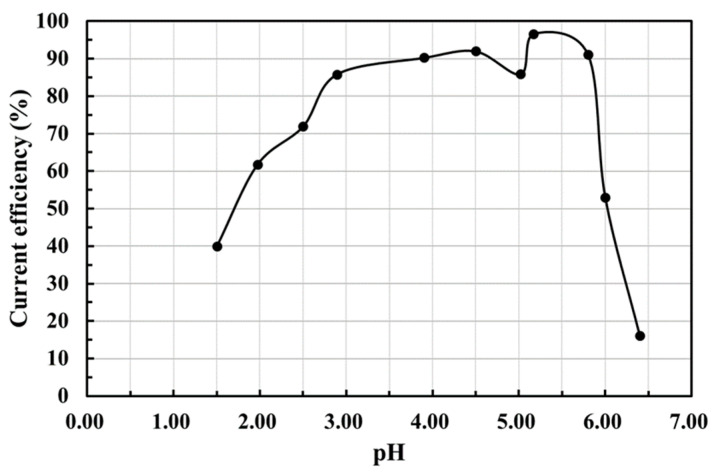
Variation of current efficiency due to change in the electrolyte’s pH value.

The electrolytic reduction of cobalt metal was conducted in an acidic sulfuric acid solution. Maintaining the electrolyte’s pH value is critical in suppressing the hydrogen ion reduction side reaction. Figure 2 shows the change in the current efficiency of cobalt metal electrolytic reduction applied at 3.4 V at room temperature for 2 h under different pH values of the electrolyte. The figure shows that a pH value lower than 3 results in a significant decrease in the current efficiency from about 85–40%. Experiment measurements show that when the acid–base value of the electrolyte is greater than 5, the pickling solution can maintain a stable current efficiency. Therefore, the best electrolytic acid–base value concluded from the experimental results is in the range of 3–5. The current efficiency of the electrolytic reduction of cobalt in this interval can be maintained at 80–90%. The electrolyte composition from the pickling of the waste catalyst is more complicated, and the range of the acid–base value of that solution should be slightly narrower than that of pure cobalt solution.

### 3.2. Influence of Current Density on the Electrolysis Efficiency

**Figure 3:**
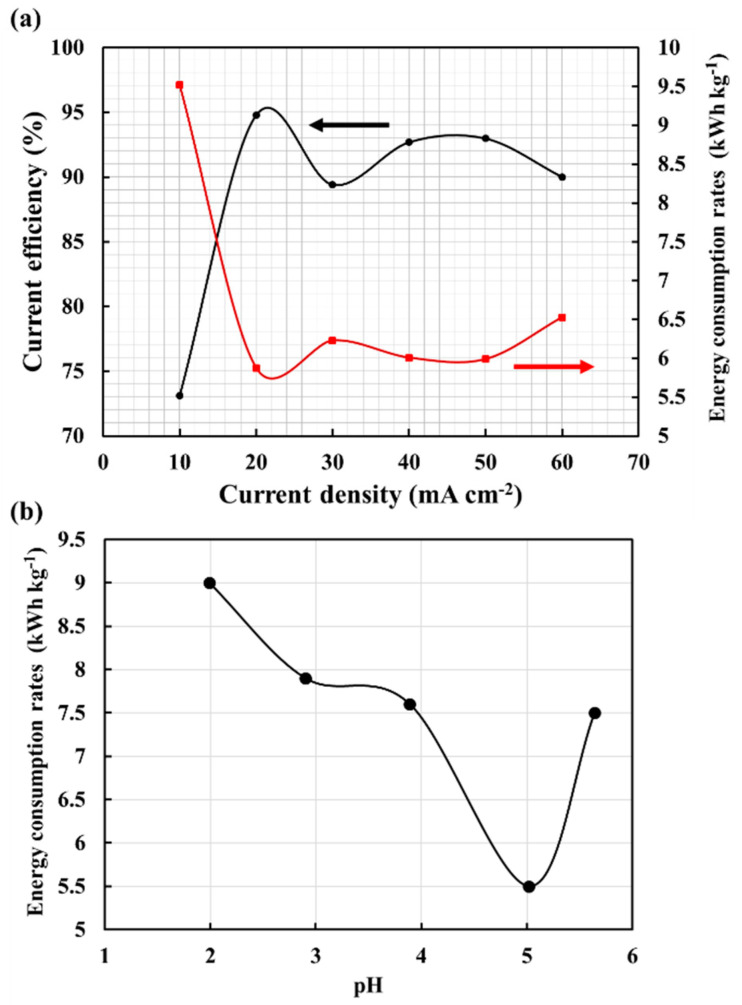
(**a**) Variation of current efficiency and energy consumption rates with current density for cobalt electrolytic reduction; (**b**) energy consumption rates under various pH values of the electrolyte with cobalt reduction operating time at 30 mA cm^−2^.

**Figure 4:**
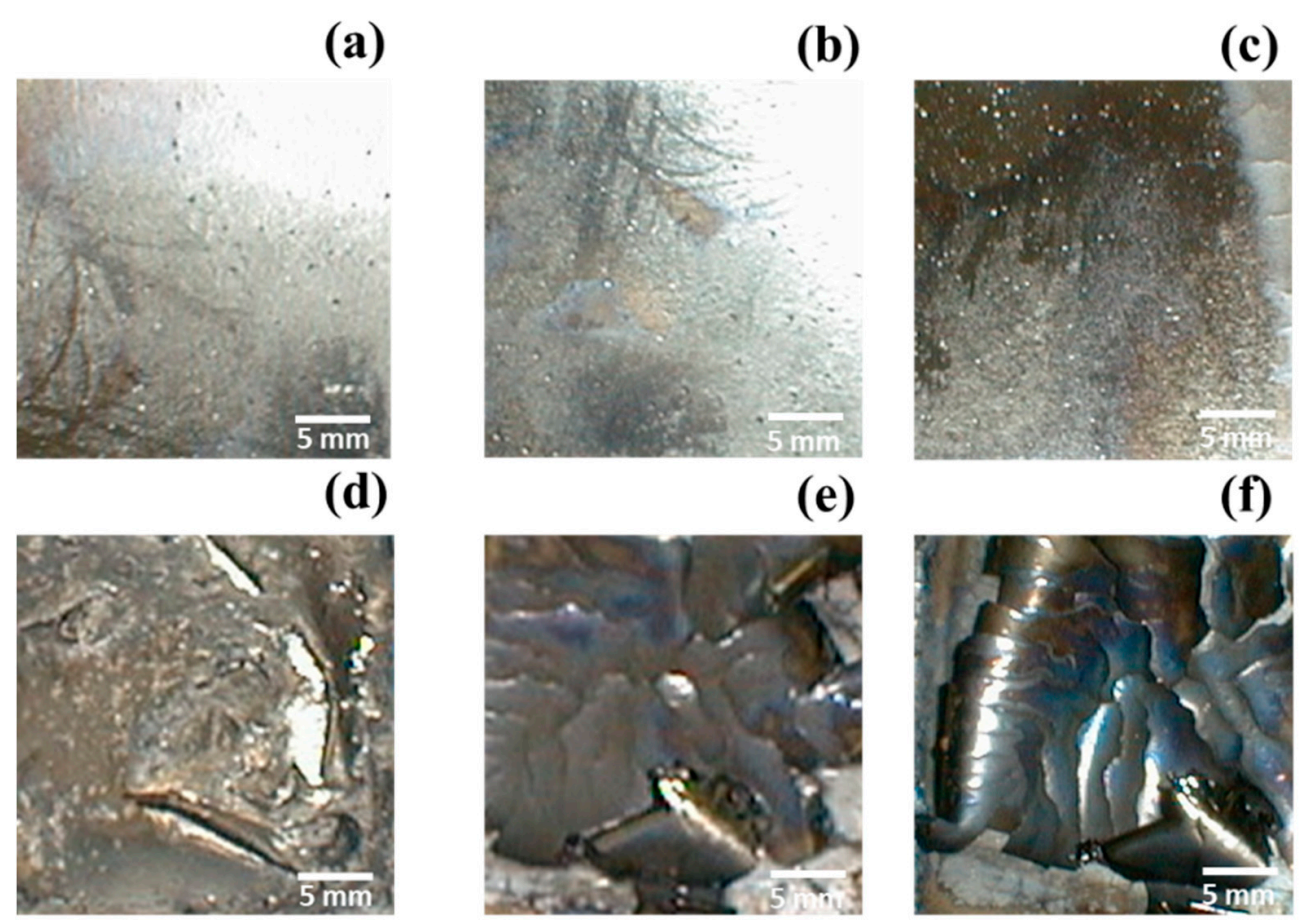
SEM surface morphology of deposited cobalt under various current density levels/values: (**a**) 10, (**b**) 20, (**c**) 30, (**d**) 40, (**e**) 50, and (**f**) 60 mA cm^−2^ of cobalt electrolytic reduction.

The more current is accepted by the system, the faster the resulting response. However, in an electrochemical system, electrodes can work effectively only at a certain current limit. If the current efficiency exceeds this range, unexpected reactions will occur on the electrodes and deteriorate their performance. Figure 3a shows the change in current efficiency and energy consumption rates for a cobalt concentration of 30 g L^−1^ with different operating current densities of 10–60 mA cm^−2^ for 2 h. It is shown that the energy consumption rates of 9.5, 5.8, 6.3, 6.0, 5.9, and 6.5 kWh kg^−1^ at the various current densities of 10–60 mA cm^−2^. Cobalt electroreduction was initiated at pH 5.64, 5.02, 3.89, 2.90, and 1.99 for 2 h with energy consumption rates of 7.5, 5.5, 7.6, 7.9, and 9.0 kWh kg^−1^, as shown in Figure 3b. The resulting quality of the cobalt deposition layer is shown in Figure 4. Figure 4 also shows that when the current density exceeds 40 mA cm^−2^ the coating becomes gray-black and easy to chip. Therefore, the experimental results conclude that for a cobalt concentration of about 30 g L^−1^ the current density should be controlled below 30 mA cm^−2^. In addition, for further decreased concentrations, it can be appropriately reduced to obtain good efficiency and quality. Das and Subbaiah studied the electrolytic reduction of pure cobalt and recommended using a current density of 10 mA cm^−2^ for bright coating [20]. Cobalt is a magnetic material, and the coating contains few impurities. Further, electrolyte contains organic substances, such as sugar, which makes the cobalt coating likely to break and fall off. However, in the traditional smelting of cobalt from cobalt ore, the current density range of 20–30 mA cm^−2^ is used due to the high concentration of cobalt.

### 3.3. pH Variation during the Electrolysis Process

**Figure 5:**
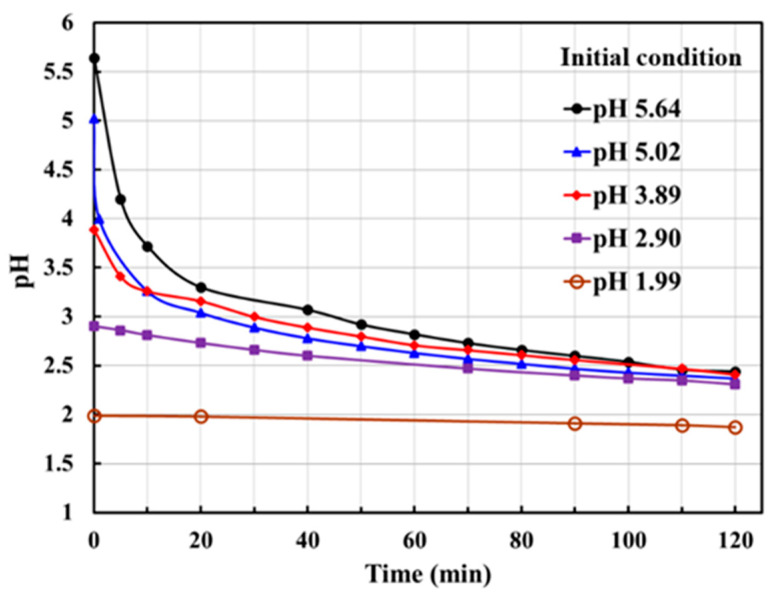
Variation in pH values of the electrolyte with cobalt reduction operating time at 30 mA cm^−2^.

In the process of cobalt electrolytic reduction from sulfuric acid pickling solution, the pH value of the electrolyte changes with time. Figure 5 shows the change in pH value of the electrolyte over the electrolysis process period at 30 mA cm^−2^. The figure shows that the pH value of the electrolyte gradually shifts toward the acidic region during the electrolysis process, which was found to negatively influence the electrolytic reduction of cobalt. This increase in acidity is due to sulfuric acid production from the reaction of anode-generated oxygen with sulfate radicals.

Another possible reason is that the hydrated salt positive-ions such as Co(H_3_O)_x_(SO_4_)_y_^−n^ may be formed from metal ions and anions, such as sulfate or carbonate, in the pickling solution. These hydrated salt ions dissociate in the presence of an electric field, which may produce sulfuric acid, increasing the acidity of the electrolyte. A previous discussion determined that for pH values lower than 3, electrolysis decreases rapidly. Therefore, to eliminate the adverse effects of the change in pH value during the electrolysis process, the electrolyte should have a controlled pH value. The pH value of 4–5 is found to be the optimum operating range.

### 3.4. Influence of Sodium Ions on the Reduction of Cobalt Electrolysis

**Figure 6:**
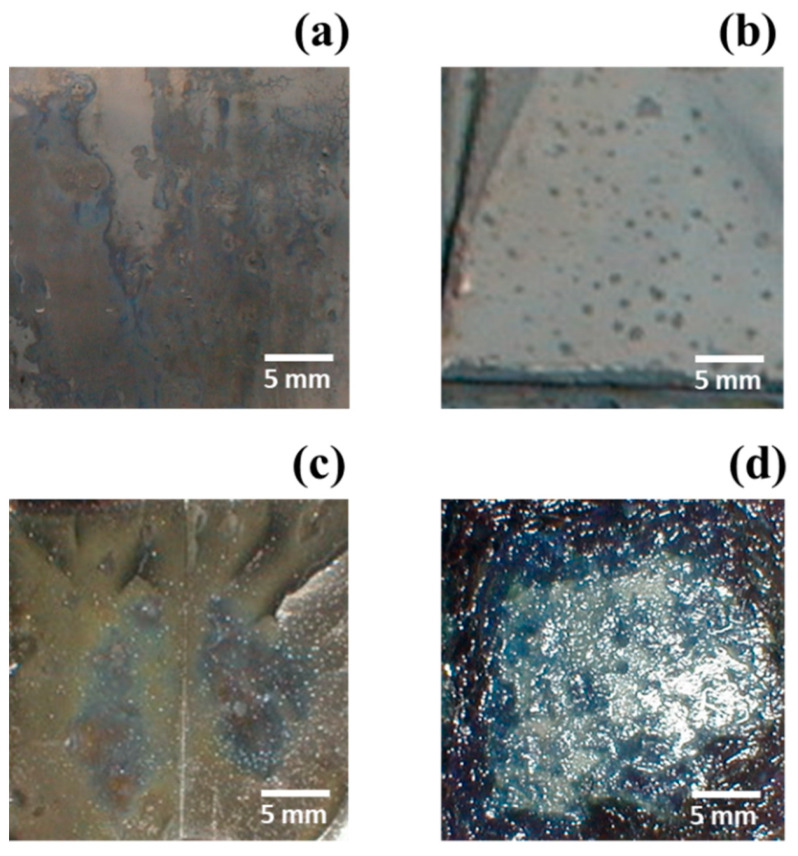
Appearance of cobalt deposition obtained by adding sodium (40 g L^−1^) and with various manganese contents of: (**a**) 7.8, (**b**) 15.7, (**c**) 21.9, and (**d**) 30.2 g L^−1^ in the pickling solution for electrolysis at 30 mA cm^−2^ for 2 h.

Fly ash from the terephthalic acid production plant contains a small amount of sodium, and different production processes have different ways of treating waste gas, which may also result in higher sodium content in the pickling solution. In addition, sodium-containing compounds are also used for baking before the pickling process. Further, to adjust the pH value in the electrolytic reduction of the pickling solution, sodium-containing compounds, such as sodium carbonate, were added to the solution. The presence of sodium in the pickling solution has a positive effect on the conductivity of the electrolyte. However, adding high sodium content (40 g L^−1^) to the sulfuric acid solution containing only cobalt ions has no adverse effect on electrolysis efficiency, and the sodium content can be as high as 0.2–0.4 wt% in the cobalt plating layer. In the presence of manganese in the pickling solution, electrolysis using a current density of 30 mA cm^−2^ for 2 h produced blue deposits on the plate, as shown in Figure 6a. The initial composition of this deposit was approximately 35 g L^−1^ Co, 6.28 g L^−1^ Na, and 7.8 g L^−1^ Mn, and their color and jelly appearance suggested metal hydroxide deposition, as shown in {Figure 6a}. The area of blue deposits expanded when the manganese content was increased by 7.8, 15.7, 21.9, and 30.2 g L^−1^. This abnormal phenomenon of blue deposits could be due to high manganese content. The cobalt concentration decreases with the electrolysis time due to an increase in [Na]/[Co] ratio with time. Therefore, the addition of sodium salt should be minimized in the program, and instead of salt, sodium carbonate ammonia can be used for adjusting the pH value.

In the electrolysis process, the cathode and anode are separated in different chambers using a separator material such as ceramics, porous plastic plates, and organic diaphragms. This is done to prevent the product of the anode reaction from disrupting the cathode reaction and to avoid the anode oxidation of the cathode-reduced product (e.g., reducing Fe^3+^ to Fe^2+^). Garritsen et al. used a diaphragm in the cobalt electrolytic reduction process to prevent oxygen generation at the anode reaction, which reduces cobalt electrolytic reduction efficiency [23]. Table 2 shows that employing organic diaphragm results in an increased current density, which gave a stable current efficiency. In the case of no diaphragm, operating in the high manganese content range is likely to produce other chemical deposition side reactions, resulting in a current efficiency even greater than 100%. This can be observed in the form of blue deposition, which is caused by side chemical reactions. From the above observations, adding a diaphragm can improve the operation and product quality of the electrolytic reduction of cobalt. However, adding a diaphragm increases the investment cost and makes the operation method complex compared with the operation without a diaphragm. It can be compensated for by implementing alternative methods such as electrolyte pH control and operating in more stable conditions; in addition, using a lower-cost nondiaphragm electrolyzer could be a solution for developing a cost-efficient process.

### 3.5. Effect of Cobalt Concentration on the Electrolysis Reduction of Cobalt

**Figure 7:**
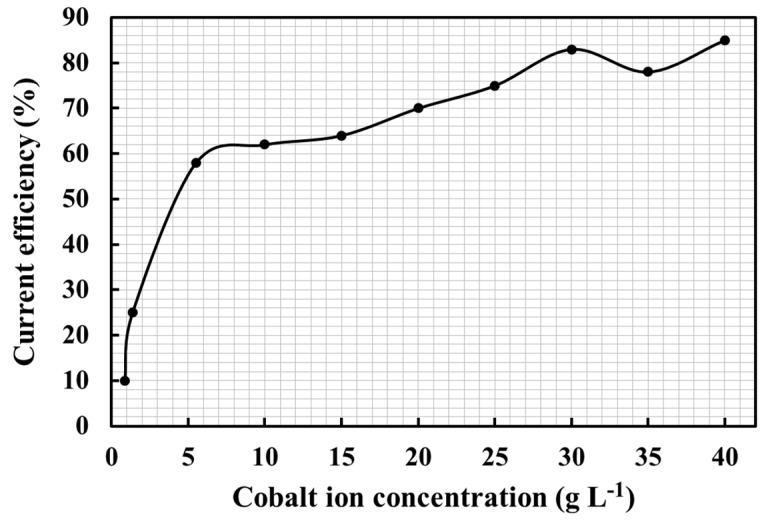
Variation of the current efficiency with a change of initial cobalt concentration in the pickling solution.

During the electrolytic reduction, cobalt is the primary reactant, and its concentration decreases steadily as the electrolysis proceeds. If the electrolysis process adopts the continuous feeding method, each part of the electrolytic cell can maintain its concentration. However, there will be a concentration difference before and after feeding, indicating a high concentration at the feeding part and a low concentration at the outlet. As the pickling solution also contains a significant quantity of other positive and negative ions, lowering the cobalt concentration reduces the current efficiency but does not result in electrolysis failure. Figure 7 shows the variation in the pickling solution’s current efficiency as a function of the initial cobalt concentration. When the cobalt concentration is reduced from 40 to 30 g L^−1^, the average current efficiency is about 80%, and a further decrease in cobalt concentration from 30 to 5.5 g L^−1^, resulting in a decrease in the average current efficiency to about 60%. The lowest average current efficiencies calculated were 25% and 10% when the cobalt concentration was reduced from 5.5 to 1.36 g L^−1^ and 1.36 to 0.85 g L^−1^, respectively. The design of a traditional flat electrode is more suitable for metal recovery from high-concentration smelting. Once the metal concentration reaches below a certain level, the mass transfer limitation current efficiency is expected to decrease.

### 3.6. Purity of Coated Cobalt

**Figure 8:**
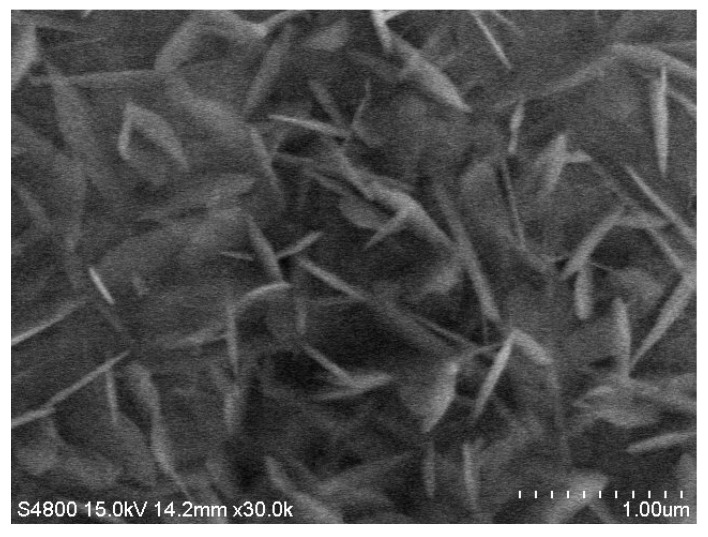
SEM surface morphology of cobalt-coated surface for electrolyte feed composition of 21.4 g L^−1^ Co, 38.2 g L^−1^ Na, and 2.02 g L^−1^ Mg, and the cobalt electrolytic reduction reaction was conducted at 30 mA cm^−2^ for 2 h.

**Figure 9:**
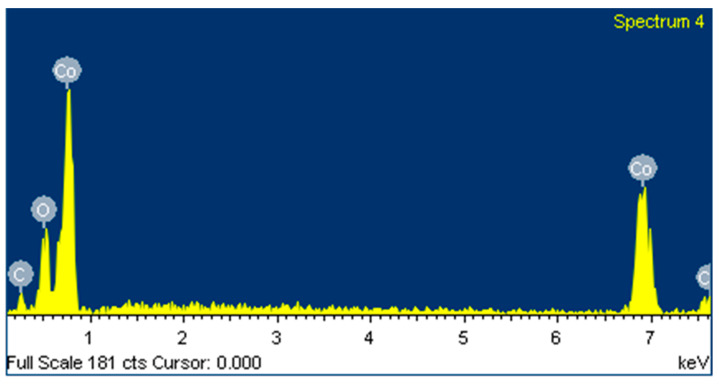
EDX spectrum of cobalt-coated surface for electrolyte feed composition of 21.4 g L^−1^ Co, 38.2 g L^−1^ Na, and 2.02 g L^−1^ Mg, and the cobalt electrolytic reduction reaction was conducted at 30 mA cm^−2^ for 2 h.

The purity of cobalt coating is significantly affected by the electrolyte’s composition under normal electrolytic reduction conditions, particularly metal impurities. Table 3 shows the purity analysis of cobalt coating under various electrolysis conditions. When the sodium content is high (40 g L^−1^), the sodium content in the coating is also high, ranging from 0.25 to 0.39 wt%, which implies that input content significantly affects the purity. In addition, if Ni and Zn were used in the input content, a high amount of about 0.95 and 5.66 wt%, respectively, was observed in the coating. Such impurities should be removed at the electrolyte purification stage. SEM and EDX were used to analyze the composition of the electrolyte feed containing 21.4 g L^−1^ Co, 38.2 g L^−1^ Na, 2.02 g L^−1^ Mg, and 30 mA cm^−2^ for cobalt electrolytic reduction reaction of 2 h. Figure 8 and Figure 9 show the surface composition analysis results. EDX spectra show that 90% cobalt crystal dendrites are mainly distributed on the metal coating surface containing a small amount of CoCO_3_. It means that sodium and magnesium impurities are contained in the internal structure of the coating. The composition of the electrolyte feed containing 21.4 g L^−1^ Co, 38.2 g L^−1^ Na, and 2.02 g L^−1^ Mg, and a current density of 30 mA cm^−2^ could recover about 99.84% of cobalt.

## 4. Conclusions

The electrochemical reduction method is used to recover cobalt metal from fly ash pickling solution of terephthalic acid waste catalyst. Cobalt electrolytic reduction is conducted at 30 mA cm^−2^ for 2 h. The average current efficiency is about 70–95%, and the energy consumption rate is about 5.5–9.5 kWh kg^−1^. The product purity can reach up to 99.84% for the electrolyte feed composition of 21.4 g L^−1^ Co, 38.2 g L^−1^ Na, and 2.02 g L^−1^ Mg. The recovery procedure can also be economically beneficial. From an economic perspective, when the cobalt concentration is below a certain level, such as 5 g L^−1^, the electrolysis operation can be stopped, and the remaining cobalt can be recovered using alternative chemical methods. Although the electrochemical reduction method can also recover low-concentration metal solutions, its electrolysis design is different from a flat electrode design. Electrodes with a high surface area per unit volume must be used, such as packed- and fluidized-bed electrodes. The important conclusions are listed as follows:

1. The acid–base value of the electrolyte significantly affects the current efficiency, and it should be maintained between pH 4 and 5.
2. The operating current density affects the quality and current efficiency of the cobalt coating, and it should be operated below 30 mA cm^−2^ for optimal results.
3. Sodium and manganese salts with low cobalt concentration and high current density result in blue hydroxide deposition. To avoid such hydroxide deposition, the process should be free from excess sodium salt.
4. Diaphragm electrolyzers are indeed efficient for good coating quality and current efficiency; however, product requirements can also be satisfied without diaphragms. To reduce investment costs and simplify equipment, diaphragm-less electrolyzers are recommended for further use.

## Figures and Tables

**Table 1:** Composition of the solution obtained from the acid immersion of waste catalyst fly ash from a terephthalic acid production plant.

Batch Number	I	II	III	IV	V	Total Electrolyte
Volume (L)	200	100	2	2	2	306
Co ions (ppm)	34,760	31,520	23,690	19,380	15,190	33,400
Mn ions (ppm)	39,360	35,840	20,380	7.56	31	37,570
Na ions (ppm)	12,640	13,215	20,000	54	124	12,712
Ca ions (ppm)	214	218	875	59	0	217
Mg ions (ppm)	39.2	38.5	2188	6	19	52.7
Fe ions (ppm)	88	51.7	3	0	0	74.4
Cu ions (ppm)	1.8	1.6	1	0	3	1.7
Zn ions (ppm)	---	---	26	250	6	1.8
Ni ions (ppm)	34.4	31.7	137	108	44	34.7
Cr ions (ppm)	10.4	8.8	---	---	20	9.8
Pb ions(ppm)	---	---	---	---	1	0.006

**Table 2:** Ending pH values of the electrolyte and current efficiency of the electrolysis with/without organic diaphragm in the electrolyzer operating at 30 mA cm^−2^ for 2 h.

Ion Concentration (g L^−1^)			Starting pH Value	Using Organic Diaphragm	Ending pH Value	Current Efficiency (%)
Co	Na	Mn				
10.4	10.7	0.0	5.01	yes	2.33	54.28
10.5	10.7	7.8	4.98	yes	2.26	94.20
10.7	10.7	7.8	5.00	no	2.29	123.96
10.6	9.9	15.7	4.99	yes	2.23	83.69
10.8	10.0	15.8	5.01	no	2.25	82.95
10.2	10.3	27.5	4.94	yes	2.29	90.03
10.5	10.1	27.3	4.98	no	2.26	89.75

**Table 3:** Purity of coated cobalt.

Input Conditions	Composition of Cobalt Electrolytic Purification Layer (%)					
	Co	Na	Mn	Mg	Ni	Zn
Co = 21.2 g L^−1^, Na = 39.5 g L^−1^, 20 mA cm^−2^	99.61	0.39				
Co = 21.5 g L^−1^, Na = 39.5 g L^−1^, 40 mA cm^−2^	99.75	0.25				
Co = 20.2 g L^−1^, Na = 30.1 g L^−1^, 30 mA cm^−2^	99.73	0.27				
Co = 10.7 g L^−1^, Na = 24.3 g L^−1^, 30 mA cm^−2^	99.63	0.37				
Co = 20.6 g L^−1^, Na = 40.1 g L^−1^, Mn = 30.2 g L^−1^, 30 mA cm^−2^	98.42	0.08	1.50			
Co = 21.4 g L^−1^, Na = 38.2 g L^−1^, Mg = 2.02 g L^−1^, 30 mA cm^−2^	99.84	0.12		0.05		
Co = 20.2 g L^−1^, Na = 38.4 g L^−1^, Ni = 512 ppm, 30 mA cm^−2^	98.95	0.10			0.95	
Co = 18.6 g L^−1^, Na = 40.6 g L^−1^, Zn = 410 ppm, 30 mA cm^−2^	93.90	0.44				5.66

## Data Availability

Not applicable.
